# Characterization of Physicochemical Properties and Microbial Communities of Tibetan Plateau Selenium-Rich Soil

**DOI:** 10.3390/microorganisms14020305

**Published:** 2026-01-28

**Authors:** Zirui Wang, Guigong Geng, Huichun Xie, Lianyu Zhou, Rui Su, Feng Qiao

**Affiliations:** 1 Key Laboratory of Tibetan Plateau Medicinal Plant and Animal Resources, School of Life Sciences, Qinghai Normal University, Xining 810008, China; 2 Academy of Agricultural and Forestry Sciences, Qinghai University, Xining 810016, China; 3 Academy of Plateau Science and Sustainability, Qinghai Normal University, Xining 810008, China; 4 Qinghai South of Qilian Mountain Forest Ecosystem Observation and Research Station, Haidong 810500, China; 5 National Forestry Grassland Qinghai Tibet Plateau Characteristic Forest and Grassland Germplasm Resources Protection and Utilization Engineering Technology Research Center, Xining 810008, China

**Keywords:** plateau region, selenium-rich soil, physicochemical parameters, selenium content, microbiomics

## Abstract

The selenium-rich area of the Tibetan Plateau is located in Qinghai Province, China, at an altitude of 2200–2500 m, with selenium content exceeding 0.3 mg/kg. This study focused on the soil selenium content, physicochemical parameters, and microbial communities of selenium-rich soils in the Ping’an selenium-rich area, as part of the Tibetan Plateau. The results showed that the total selenium contents in both the Ping’an and Guide areas were significantly higher than in the control, ranging from 624.56 µg/kg to 727.48 µg/kg in Ping’an and reaching 721.74 µg/kg in Guide. Correlation analysis revealed that organic selenium content was significantly positively correlated with total phosphorus (*p* < 0.05), effective phosphorus (*p* < 0.01), and available potassium (*p* < 0.05) contents. Within the bacterial community, organic selenium content showed significant positive correlations with the abundance of *Arthrobacter crystallopoietes* (*p* < 0.01), *Nitrosospira briensis* (*p* < 0.01), and unclassfied *Rhodobacteraceae* (*p* < 0.01). Total selenium content was significantly negatively correlated with the abundance of *Tepidisphaera mucosa* (*p* < 0.01). RDA analysis indicated that total potassium contributed the most (30.52%), followed by total nitrogen (21.47%) and total phosphorus (15.07%). In the fungal community, organic selenium content was significantly positively correlated with the abundance of *Tausonia pullulans* (*p* < 0.01), *Botryotrichum domesticum* (*p* < 0.01), *Preussia flanaganii* (*p* < 0.05), and *Enterocarpus grenotii* (*p* < 0.01). RDA analysis showed that total phosphorus contributed the most (27.30%), followed by total potassium (21.70%) and total nitrogen (14.86%). The findings provide a scientific basis for understanding soil physicochemical properties and microbial diversity in plateau selenium-rich regions and lay a foundation for the isolation and utilization of dominant microbial species in these soils.

## 1. Introduction

Selenium is an essential trace element for humans [1,2]. It functions as a cofactor for antioxidant enzyme systems within the body [3], plays a crucial role in regulating inflammatory processes [4], and is an indispensable element for the normal life functions of both humans and animals [5,6]. Concurrently, selenium is also an essential micronutrient for animals and plants [7], with its content in soil significantly influencing its uptake by these organisms [8]. In nature, selenium is typically absorbed by the living cells of microorganisms, plants, animals, and humans in various inorganic forms [9], such as selenate, selenite, elemental selenium, and selenides [1]. In China, the distribution of selenium varies widely, with concentrations ranging from 0.007 to 25.42 mg/kg [10]. The eastern Qinghai region is a significant enrichment zone for selenium-rich resources in China, where 9244 km^2^ of selenium-rich resources have been identified [11]. This includes 544 km^2^ in the Qaidam Basin and 8700 km^2^ in Haidong and Haibei. The concentrated contiguous areas of selenium-rich soil in Haidong and Haibei are primarily located in the Ping’an District of Haidong, Laji Mountain, Menyuan, and Gangcha. Among these, the lacustrine sedimentary type of selenium-rich soil in Ping’an holds the most promising development potential [12].

Soil serves as the primary source of human selenium intake, and its selenium content and bioavailability are significantly influenced by soil physicochemical properties [13]. Studies indicate that soil selenium content is significantly affected by soil organic matter, total potassium, iron oxides, and aluminum oxides, with contribution rates of 15%, 6%, 20%, and 22%, respectively [14]. Other research shows that selenium is negatively correlated with pH and total potassium, while it is significantly positively correlated with cation exchange capacity (CEC), soil organic matter (SOM), humus, total nitrogen (TN), and total phosphorus (TP) [15]. However, the effects of soil texture, organic matter, and pH on selenium and its bioavailability are complex and can be opposing; for instance, factors like clay texture, high organic matter, and low pH can promote selenium accumulation in soil, but the association of selenium with organic matter also limits its mobility and availability to plants [16,17]. Notably, the influence of pH on selenium absorption diminishes with increasing soil organic matter content [18].

Among the various migration and transformation pathways of selenium, microbial transformation is particularly important and represents the primary form of selenium transformation in the soil environment [19]. As a vital trace element, selenium not only enhances plant resistance to stresses but also modulates and optimizes the rhizosphere microbial community [20,21,22]. Research shows that changes in the microbial community are strongly linked to soil environmental indices and selenium speciation, influencing the bioavailability of pollutants such as heavy metals [23,24]. Specifically, selenium primarily enriches Proteobacteria and Actinobacteria [25]. Selenium-related bacteria, such as Bacillus and Dyella, dominate in dryland soils characterized by higher available selenium and lower contents of water, total organic carbon (TOC), ammonium nitrogen, and nitrite [26]. Key microorganisms, including *Pseudomonas* and *Bacillus*, exhibit diverse mechanisms for selenium reduction and play a significant role in the global selenium cycle [27]. *Agrobacterium* sp. T3F4, isolated from selenium-rich soil, can oxidize elemental selenium into selenite under pure culture conditions [28]. Experiments have demonstrated that three selenium-oxidizing bacteria (LX-1, LX-100, T3F4) significantly increase selenium content in plants and soil, raising the selenium content in the aboveground parts of plants by 330.8%, 309.5%, and 724.3%, respectively, and increasing soil available selenium by 38.4%, 20.4%, and 24.0% compared to the control [29]. Furthermore, arbuscular mycorrhizal fungi (AMF) play a vital role in enhancing selenium uptake in crops [30]. AMF agents can improve selenium bioavailability by enhancing soil acid phosphatase activity and promoting root vitality [31]. It is noteworthy that, despite the various positive effects of selenium on microorganisms, the addition of 10 ppm selenium significantly reduces bacterial alpha-diversity [32].

However, there are no reports on the selenium content, physicochemical properties, and microbial diversity of the selenium-rich soil in the Ping’an area of the Qinghai-Xizang Plateau, and no research has been conducted on the relationship between altitude and selenium accumulation. Meanwhile, the relationship between the physical and chemical properties of the soil in plateau areas and the accumulation of selenium in the soil has not yet been studied. In this study, the selenium content, physicochemical properties, microbial community structure, and diversity of the soil in the Ping’an area, a high-altitude selenium-rich region, were investigated. The dominant microbial community rich in selenium was screened, and the correlation between the dominant microbial community and soil physicochemical indicators was explored. The research results reveal the relationship between the physicochemical properties and dominant microbial communities of selenium-rich soils in high-altitude areas, providing a scientific basis for the screening, isolation, and development of dominant microbial communities in selenium-rich areas.

## 2. Materials and Methods

### 2.1. Study Area

In late June 2025 (during the plant-growing season), field investigations and soil sample collection were carried out in the study area. This study conducted sampling in the Ping’an District, Guide County, and Datong District of Qinghai Province (Ping’an site: 102°5′ E, 36°27′ N; Guide site: 101°25′ E, 36°2′ N; Datong site: 101°41′ E, 36°55′ N) (Table 1). For each plot, three 5 m × 5 m subplots were established. Based on the principle of random and multi-point sampling, soil samples with a depth of approximately 20 cm were collected from each large plot. After the soil samples from the same area were thoroughly mixed, one sample was obtained. Each sample was repeated three times.

The sampling areas are all located at the northeastern edge of the Qinghai-Xizang Plateau, in the Yellow River and Huangshui River valleys. The altitude ranges from 2200 to 2400 m. This area has a warm temperate semi-arid climate, with an average annual temperature of 4 to 8 °C and an average annual evaporation of 500–2000 mm (Table 2). During the soil formation process in this area, the soil parent material has a significant controlling effect on the selenium (Se) content. Geological background studies indicate that the coal-bearing sedimentary rocks, intermediate-basic volcanic rocks, and Paleogene saline lake red mudstones in the region are the main sources of selenium-rich soil [33]. In addition, the adsorption effect of soil organic matter in this area is also an important factor leading to the enrichment of selenium in the surface layer.

**Table 1:** Basic information sheet of soil sampling sites.

Serial Number	Sampling Location	Altitude/m	East Longitude	North Latitude	Soil Type
CK	Datong District	2400.0	101°41′8″	36°55′37″	Haplic Luvisols [34]
GD	Guide County	2225.8	101°25′42″	36°2′29″	Calcic Kastanozems [34]
PA1	Ping’an District	2256.7	102°5′56″	36°27′13″	Haplic Luvisols [34]
PA2	Ping’an District	2296.2	102°5′57″	36°27′15″	Haplic Luvisols [34]
PA3	Ping’an District	2392.46	102°5′54″	36°27′8″	Haplic Luvisols [34]

**Table 2:** Climatic characterization of the soil sampling sites.

Serial Number	Average Annual Precipitation	Average Annual Temperature	Annual Sunshine Duration	Annual Average Evaporation
CK	450~800 mm	4~5 °C	2500~2600 h	1500~2000 mm
GD	250~350 mm	7~8 °C	2700~3000 h	1500~2000 mm
PA1	300~400 mm	6~7 °C	2600~2800 h	1500~1800 mm
PA2	300~400 mm	6~7 °C	2600~2800 h	1500~1800 mm
PA3	300~400 mm	6~7 °C	2600~2800 h	1500~1800 mm

### 2.2. Measurement of Soil Physicochemical Properties

Selenium speciation analysis involved determining total selenium after complete digestion and inorganic selenium following selective extraction, both measured by atomic fluorescence spectrometry. Organic selenium was calculated as the difference between the total and inorganic selenium contents [35,36].

Total phosphorus was measured using sodium hydroxide fusion [37]. This process converted all phosphorus forms into soluble orthophosphates, which were subsequently determined by the molybdenum blue method using spectrophotometric detection. Effective phosphorus was extracted with a sodium bicarbonate solution [37], which effectively dissolved labile phosphorus pools in calcareous soils. The extracted phosphorus was quantified colorimetrically via the molybdenum blue method. Organic matter was quantified using the dichromate wet oxidation method with external oil bath heating at 180 °C [37]. Soil organic matter was oxidized by a potassium dichromate–sulfuric acid solution, and the unconsumed oxidant was titrated with ferrous sulfate to calculate the organic matter content. Total nitrogen was determined by sulfuric acid digestion with catalysts [37]. Potassium sulfate and copper sulfate were added to concentrated sulfuric acid during high-temperature digestion, facilitating the conversion of all nitrogen forms to ammonium nitrogen. The resulting ammonium sulfate digest was then analyzed by a continuous flow analyzer. Soil pH was measured potentiometrically using a combined glass electrode [37], which detected the hydrogen ion activity in the soil solution and displayed the corresponding pH value. Alkaline hydrolysis of nitrogen was determined by the alkaline hydrolysis diffusion method [37]. Soil was treated with 1.8 mol·L^−1^ sodium hydroxide, and the liberated ammonia was absorbed in boric acid, followed by titration with standard acid. Total potassium was analyzed by flame photometry following sodium hydroxide fusion [37]. The characteristic light emission from potassium atoms in a flame was measured, and the intensity was compared against standard solutions for quantification. Available potassium was measured by flame photometry using ammonium acetate extraction [37]. The potassium concentration in the extract was determined by measuring its characteristic emission intensity at 766.5 nm. Exchangeable calcium and magnesium were extracted with ammonium chloride in 70% ethanol (pH 8.5) and quantified directly by atomic absorption spectrometry [38].

### 2.3. Determination of Soil Microbial Community Structure

The study of microbial diversity uses operational taxonomic units (OTUs) as molecular markers to investigate the taxonomic classification and species composition of microorganisms in environmental samples. This research is mainly achieved by targeting the conserved regions of nucleic acid sequences encoding ribosomal RNA (rRNA). For bacteria, the analysis is mainly based on the 16S rRNA gene region (primers: F: AGRGTTTGATYNTGGCTCAG; R: TASGGHTACCTTGTTASGACTT); for fungi, it mainly focuses on the internal transcribed spacer (ITS) region (primers: F: CTTGGTCATTTAGAGGAAGTAA; R: TCCTCCGCTTATTGATATGC).

Library Preparation and Sequencing: Total genomic DNA was extracted from the samples, and PCR amplification was performed using specific primers with unique barcodes synthesized based on full-length sequences. The resulting products were purified, quantified, and normalized to construct SMRTbell sequencing libraries according to the PacBio SMRTbell Preparation Protocols (https://www.pacb.com/support/documentation/, (accessed on 20 December 2025)). The prepared libraries were then sequenced using the PacBio Sequel II system (Pacific Biosciences, Menlo Park, CA, USA; https://www.pacb.com/products/sequencing-platforms/sequel-ii-system/, (accessed on 20 December 2025)), yielding raw data in BAM format. High-quality Circular Consensus Sequencing (CCS) files were subsequently generated using the SMRT Link analysis software (version 13.0, (accessed on 20 December 2025)). Based on the barcode sequences, data from different samples were demultiplexed and converted into FASTQ format for downstream analysis.

Data Preprocessing: After exporting the raw PacBio data as CCS files (CCS reads are obtained using the SMRT Link tool (version 13.0, (accessed on 20 December 2025)) provided by PacBio, Menlo Park, CA, USA), the main steps are as follows. CCS Demultiplexing: Use lima v1.7.0 (https://github.com/PacificBiosciences/barcoding/, (accessed on 20 December 2025)) to demultiplex CCS reads based on barcodes, obtaining Raw CCS sequence data. CCS Filtering: Use cutadapt v1.9.1 to identify and remove primer sequences and perform length filtering, obtaining Clean CCS sequences without primer sequences. Chimera Removal: Use UCHIME v4.2 (http://drive5.com/uchime, (accessed on 20 December 2025)) to identify and remove chimeric sequences, obtaining Effective CCS sequences.

Information Analysis Content: Using the Usearch software (version 11.0), we clustered the Tags at a 97% similarity level, obtained OTUs, and performed taxonomic annotation on the OTUs based on the Silva 138.2 (http://www.arb-silva.de/, (accessed on 20 December 2025)) (bacteria) and UNITE (version 10.0, http://unite.ut.ee/, (accessed on 20 December 2025)) (fungi) taxonomic databases. The Alpha diversity index of the samples was evaluated using the Mothur (version v.1.30) software (http://www.mothur.org/, (accessed on 20 December 2025)). The Beta diversity (species diversity) analysis was conducted using the QIIME software (version 2024.2) to compare the similarities in species diversity among different samples. The Beta diversity analysis mainly employed four algorithms for calculating the distances between samples: binary jaccard, bray curtis (https://en.wikipedia.org/wiki/Bray-Curtis_dissimilarity, (accessed on 20 December 2025)), weighted unifrac (https://en.wikipedia.org/wiki/UniFrac, (accessed on 20 December 2025)) (for bacteria), and unweighted unifrac (https://en.wikipedia.org/wiki/UniFrac, (accessed on 20 December 2025)) (for bacteria). These algorithms were used to obtain the values between the samples.

### 2.4. Statistical Analysis

Principal coordinate analysis (PCoA) was performed to assess the similarity of microbial community structures among different sampling sites. Based on Bray–Curtis distance matrices derived from bacterial and fungal ASV abundance data, PCoA reduced the multidimensional data into two principal coordinates for visualization, wherein the spatial proximity between sample points reflects the degree of similarity in community composition.

Redundancy analysis (RDA) was conducted to evaluate the relationship between soil physicochemical properties and microbial community structure. Environmental variables, including total selenium, organic selenium, pH, organic matter, total nitrogen, total phosphorus, total potassium, available phosphorus, available potassium, and exchangeable calcium and magnesium, were included as explanatory variables. The significance of each environmental factor was tested using permutation tests, and the proportion of community variance explained by each factor was calculated. All multivariate analyses were carried out using the vegan package (version 2.6-4; https://CRAN.R-project.org/package=vegan, accessed on 20 December 2025) in R (version 4.4.3).

The Pearson correlation analysis was conducted using the Origin 2021 software (OriginLab Corporation, Northampton, MA, USA; https://www.originlab.com/, accessed on 20 December 2025) to evaluate the relationship between soil physical and chemical properties and the composition of microbial communities. The statistical analysis included the calculation of means and standard errors, which were all performed using SPSS 27 (https://www.ibm.com/cn-zh/products/spss, (accessed on 20 December 2025)) software. The bar charts of various soil physical and chemical indicators were drawn using Microsoft Excel 2024 (https://www.microsoft.com/zh-cn/microsoft-365/excel, (accessed on 20 December 2025)), and error lines were marked.

## 3. Results

### 3.1. Analysis of Soil Selenium Content

**Figure 1:**
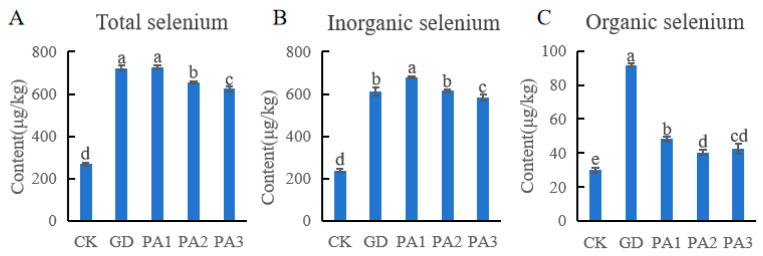
The selenium content of soil. (**A**) The total selenium content in the soil of different regions. (**B**) The inorganic selenium content in the soil of different regions. (**C**) The organic selenium content in the soil of different regions. Different lowercase letters (a, b, c, d and e) indicate significant differences between groups at the *p* < 0.05 level according to Duncan’s multiple range test.

The changes in selenium content in soils from five different regions are shown in Figure 1. The total selenium and inorganic selenium contents in the four different areas, GD, PA1, PA2, and PA3, were significantly higher than those in the control CK area. The total selenium contents in the four different areas ranged from 624.56 to 727.47 µg/kg, while that in CK was 268.04 µg/kg. The inorganic selenium contents in the four different areas ranged from 582.07 to 679.28 µg/kg, while that in CK was 238.20 µg/kg. The total selenium content and inorganic selenium content in PA1area reached the peak. In contrast, the organic selenium content, with 91.67 µg/kg, in the GD area was significantly higher than in other areas, with 40.27–48.20 µg/kg. In the soil of the planting area, inorganic selenium is the dominant form of total selenium, with an average proportion as high as 91%. According to the standard set by the Ministry of Natural Resources of the People’s Republic of China, which defines soil with selenium content ≥ 0.3 mg/kg as selenium-rich soil [39], except for CK, the other four investigated sites have all reached the selenium-rich level.

These results indicated that the distribution and content of soil selenium forms were not only influenced by environmental factors, such as temperature and humidity driven by the altitude gradient, but are also closely related to the inherent physical and chemical properties of the soil. The PA1 area may become a selenium enrichment center due to its special parent material and higher selenium adsorption and fixation capacity, while the relatively abundant organic matter in the GD area may promote the microbial-mediated transformation of selenium forms and the accumulation of organic selenium.

### 3.2. Analysis of Soil Physicochemical Properties

**Figure 2:**
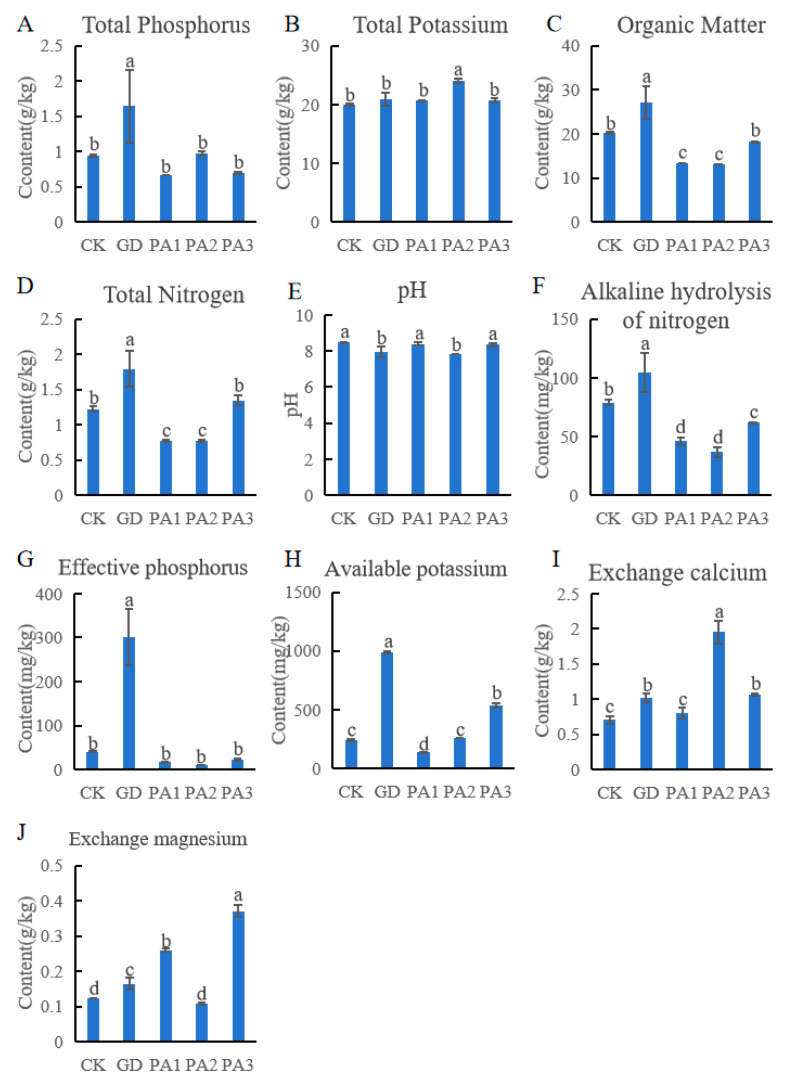
Physicochemical properties of the soil. (**A**) The total phosphorus content in the soil of different regions. (**B**) The total potassium content in the soil of different regions. (**C**) The organic matter content in the soil of different regions. (**D**) The total nitrogen content in the soil of different regions. (**E**) The pH in the soil of different regions. (**F**) The alkaline hydrolysis of nitrogen content in the soil of different regions. (**G**) The effective phosphorus content in the soil of different regions. (**H**) The available potassium content in the soil of different regions. (**I**) The exchange calcium content in the soil of different regions. (**J**) The exchange magnesium content in the soil of different regions. Different lowercase letters (a, b, c and d) indicate significant differences between groups at the *p* < 0.05 level according to Duncan’s multiple range test.

Soil physical and chemical properties vary significantly among different regions, and even within the same region, soil properties at different altitudes show significant heterogeneity (Figure 2). The total phosphorus, organic matter, total nitrogen, alkaline hydrolysis of nitrogen, effective phosphorus, and available potassium in the GD region were high. The total phosphorus content in GD was 1.65 g/kg, while that in the four different areas was 0.66–0.97 g/kg. The organic matter content in GD was 27.11 g/kg, while that in the four different areas was 13.07–20.25 g/kg. The total nitrogen content in GD was 1.79 g/kg, while that in the four different areas was 0.77–1.35 g/kg. The effective phosphorus content in GD was 300.59 g/kg, while that in the four different areas was 9.39–40.23 mg/kg. The available potassium content in GD was 985.90 mg/kg, while that in the four different areas was 138.30–537.88 g/kg.

The total potassium and exchangeable calcium contents in the PA2 region were high. The exchangeable magnesium content in the PA3 region was high. The total potassium content in PA2 was 24.09 g/kg, while that in the four different areas was 19.98–20.93 g/kg. The exchangeable calcium content in PA2 was 1.95 g/kg, while that in the four different areas was 0.71–1.07 g/kg.

Generally, the soil in the five regions was mostly neutral to slightly alkaline, with pH value of 7.85–8.49.

Briefly, many physicochemical indicators in the Guide region have higher concentrations than in other areas.

### 3.3. Correlation of Soil Physicochemical Properties

**Figure 3:**
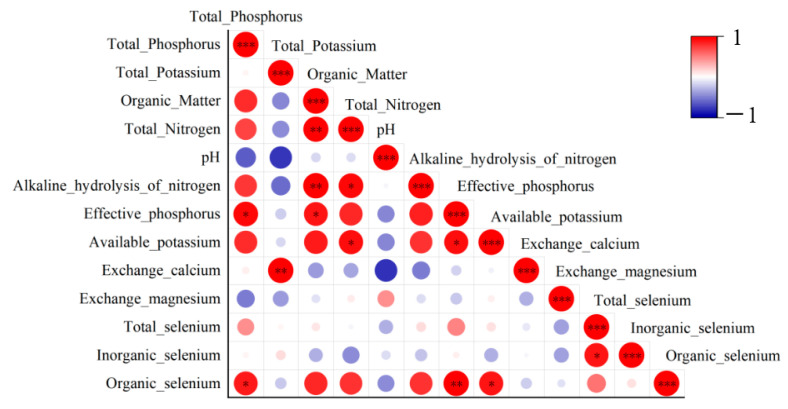
Heatmap of the correlation of physicochemical properties of soil. (* *p* < 0.05, ** *p* < 0.01, *** *p* < 0.001).

The correlations among 13 physical and chemical properties of the soil were analyzed (Figure 3). There were 12 significantly positive correlations among them, while there were no significantly negative correlations. Inorganic selenium was significantly positively correlated with total selenium (*p* < 0.05, r = 0.90). Organic selenium was significantly positively correlated with total phosphorus (*p* < 0.05, r = 0.88), effective phosphorus (*p* < 0.01, r = 0.99), and available potassium (*p* < 0.05, r = 0.89).

Organic matter was significantly positively correlated with total nitrogen (*p* < 0.01, r = 0.97), alkaline hydrolysis of nitrogen (*p* < 0.01, r = 0.99), and effective phosphorus (*p* < 0.01, r = 0.88). Total potassium was significantly positively correlated with exchangeable calcium (*p* < 0.01, r = 0.98). Effective phosphorus was significantly positively correlated with total phosphorus (*p* < 0.05, r = 0.94). Total nitrogen was significantly positively correlated with alkaline hydrolysis of nitrogen (*p* < 0.05, r = 0.94), and available potassium (*p* < 0.05, r = 0.91). Effective phosphorus was significantly positively correlated with available potassium (*p* < 0.05, r = 0.90).

### 3.4. OTU Statistical Analysis of Soil Microorganisms

**Figure 4:**
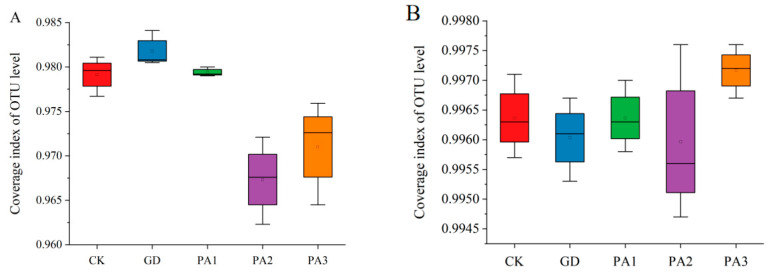
OTU statistical analysis of soil microorganisms. (**A**) Bacterial community coverage. (**B**) Fungal community coverage.

OTU statistical analysis was conducted on soil samples from five districts, with three repetitions for each district. In the detection of the bacterial community in soil (Figure 4A), a total of 416,669 quality control sequences were obtained from all samples, with an average of 27,778 sequences per sample. A total of 36,148 OTUs were identified through clustering and classification, belonging to 2 kingdoms, 34 phyla, 68 classes, 197 orders, 377 families, 807 genera, and 1530 species. The coverage of all samples was above 0.95 (Figure 4A), indicating that the sequencing results can accurately reflect the true structure of the bacterial community in soil.

In the detection of the fungal community in soil (Figure 4B), a total of 521,486 quality control sequences were obtained from all samples, with an average of 34,766 sequences per sample. A total of 9026 OTUs were identified through clustering and classification, belonging to 1 kingdom, 15 phyla, 49 classes, 113 orders, 220 families, 444 genera, and 701 species. The coverage of all samples was above 0.95 (Figure 4B), indicating that the sequencing results can accurately reflect the true structure of the fungal community in soil.

### 3.5. Analysis of the Alpha-Diversity of Soil Microorganisms

**Figure 5:**
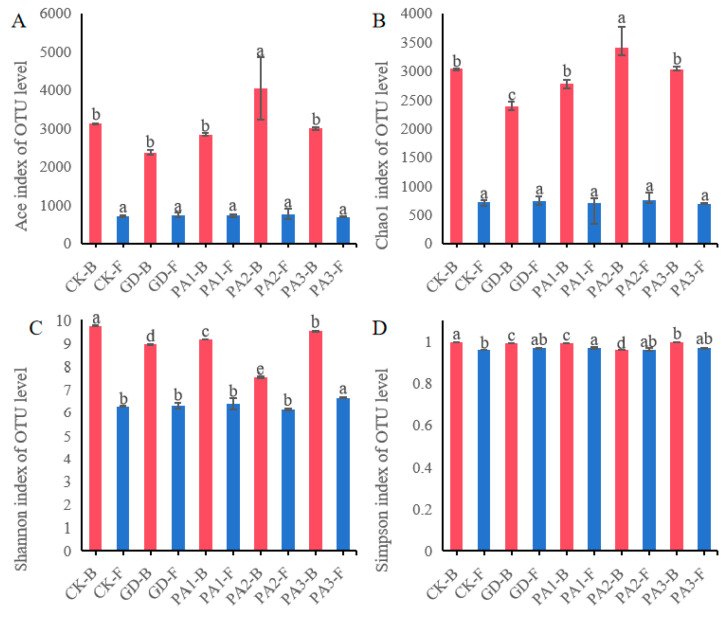
The alpha-diversity index of soil microorganisms (bacteria in red and fungi in blue, different lowercase letters (a, b, c, d and e) indicate significant differences between groups at the *p* < 0.05 level according to Duncan’s multiple range test.). (**A**) Ace index at the OTU level. (**B**) Chao1 index at the OTU level. (**C**) Shannon index at the OTU level. (**D**) Simpson index at the OTU level.

The alpha-diversity indices of fungal and bacterial communities in different regions of the soil were analyzed (Figure 5). Among them, the Ace index and Chao1 index are key indicators reflecting the richness of fungal and bacterial communities. The results showed that in the five sampling points, the Ace (Figure 5A) and Chao1 (Figure 5B) indices of the bacterial community in the PA2 region were the highest, indicating that the soil environment in this region may be more conducive to the colonization of bacteria and the coexistence of multiple bacterial groups. The Ace and Chao1 indices of the bacterial community at the GD sampling point were significantly lower than those at other sampling points, suggesting that there might be certain stress factors in the soil that inhibit bacterial amplification or limit resource utilization. In contrast, the Ace and Chao1 indices of the fungal community in all five regions showed no significant differences, indicating that the species richness of the fungal community is less affected by regional environmental variations and has relatively strong stability. The Shannon index and the Simpson index are important indicators for evaluating microbial community diversity. The Shannon (Figure 5C) index of the bacterial community at the PA2 sampling point was significantly lower than that in other regions, indicating that the species distribution within the community was uneven, and there might be a phenomenon of a few dominant bacterial groups occupying a dominant position. The Shannon index of the bacterial community at the CK sampling point was significantly lower than that in other regions, further reflecting that the bacterial community structure in this area was simple, with concentrated species distribution, and might be subject to the screening effect of specific environmental factors. The Shannon index of the fungal community was very close among the sampling points, with the PA3 region slightly higher than the other regions, indicating that the uniformity of the fungal community was relatively consistent in different habitats, and environmental disturbances had a limited impact on it. On the other hand, the Simpson index (Figure 5D) of the soil at all sampling points was at a relatively high level and close to each other, indicating that there were obvious dominant groups in each soil microbial community, and their concentration degree was not significantly different among different regions. This result suggests that although the richness and diversity of bacterial communities fluctuated significantly among regions, the construction of microbial communities in local environments was always dominated by a few key species.

Overall, the alpha-diversity of bacterial communities in the soil shows a high sensitivity to spatial heterogeneity, while fungal communities demonstrate a stronger ability to buffer environmental changes in terms of richness and evenness. This result indicates that the two microbial communities may adopt different resource utilization strategies and ecological niche adaptation mechanisms in the same habitat.

### 3.6. Composition of Soil Microbial Communities

**Figure 6:**
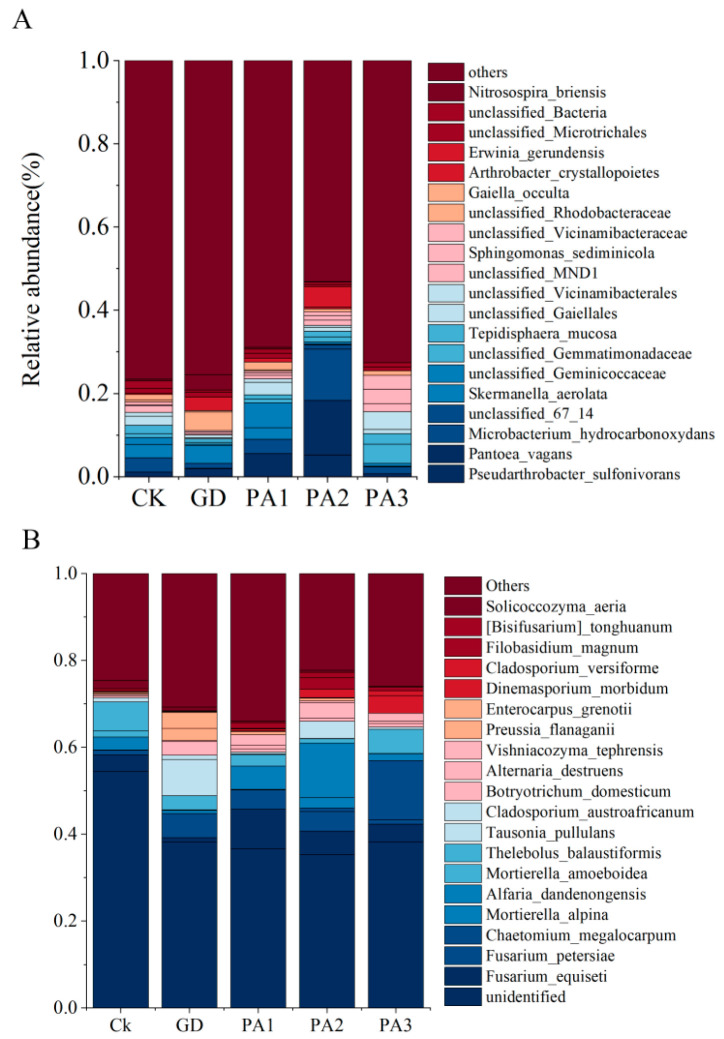
The composition of the microbial community in the soil. (**A**) The composition of the bacterial community in the soil (species). (**B**) The composition of fungal communities in the soil (species).

At the species level, an analysis was conducted on the top 20 bacterial species in terms of relative abundance across different sampling sites (Figure 6A). The results revealed that representative taxa included *Gaiella occulta*, *Sphingomonas sediminicola*, and *Arthrobacter crystallopoietes*, among others. At the phylum level, these bacteria primarily belonged to Actinobacteria, Proteobacteria, and Gemmatimonadota, with Actinobacteria being the most dominant group. Members of Actinobacteria typically possess strong capabilities for organic matter degradation and secondary metabolite synthesis, playing a crucial role in soil element cycling and the maintenance of ecosystem functions.

In terms of fungi, the top 20 species in terms of relative abundance at the species level (Figure 6B) showed that two species belonged to the genus Fusarium, namely, *Fusarium petersiae* and *Fusarium equiseti*, while two other species belonged to the genus *Mortierella*, including *Mortierella alpina* and *Mortierella amoeboidea*, making these the most frequently occurring genera in the community. The remaining species were distributed across genera such as *Cladosporium*, *Tausonia*, *Thelebolus*, *Alfaria*, and *Chaetomium*, among others. At the phylum level, these fungi primarily belonged to Ascomycota and Basidiomycota, with Ascomycota being the significantly dominant group, encompassing the majority of the species. This aligns with its status as the most species-rich group within the fungal kingdom and reflects its broad adaptability and functional importance in soil ecosystems.

From the species-level perspective, the structure of the soil microbial community exhibits distinct distribution characteristics. The top 20 dominant species of the bacterial community mainly belong to the Actinobacteria and Bacteroidetes phyla, with specific species of the *Gaiella* genus and *Filimonas* genus serving as the core. The dominant composition of the fungal community spans the Ascomycota and Basidiomycota phyla, with representative species belonging to the *Fusarium* genus and *Mortierella* genus. This structure reflects the distinct ecological niches occupied by fungi and bacteria in soil ecosystems and their complementary functions. Fungi likely play important roles in organic matter decomposition and the transformation of recalcitrant substances, while bacteria contribute significantly to element cycling and secondary metabolite synthesis. Together, they form a complex network of microbial interactions. This community composition further indicates that the microbial community structure is shaped by both vegetation type and soil environmental factors and is closely related to its ecological functions.

### 3.7. Soil Microbial Community PCoA

**Figure 7:**
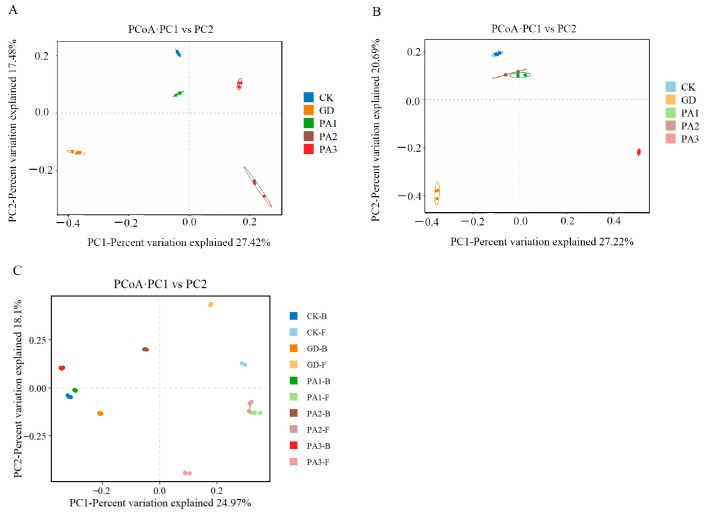
Principal coordinate analysis (PCoA) diagram of soil microbial. (**A**) Principal coordinate analysis (PCoA) diagram of bacterial community composition. (**B**) Principal coordinate analysis (PCoA) diagram of fungal community composition. (**C**) Principal coordinate analysis (PCoA) diagram of bacterial and fungal community composition.

Principal coordinate analysis (PCoA) was used to evaluate the similarity of soil microbial community compositions across different sampling sites (Figure 7). In the ordination plot, the spatial distance between sample points reflects the degree of similarity in community structure, with closer distances indicating greater similarity in community composition. In the bacterial community (Figure 7A), the five sampling points were relatively dispersed in the PCoA plot, indicating significant heterogeneity in bacterial community structure among the different sampling sites and greater sensitivity to microenvironmental differences at each site. Among them, sample points CK, PA1, and PA3 were slightly closer in the ordination plot, suggesting that despite the overall significant differences, these three sampling points still share some core bacterial taxa or are influenced by certain common environmental factors.

In the fungal community (Figure 7B), sample points CK, PA1, and PA2 clustered closely, while they were relatively distant from PA3 and GD, indicating that the fungal community structures of the first three sampling points were relatively similar and may be regulated by similar ecological processes or environmental factors. In contrast, PA3 and GD may have developed unique fungal communities due to local soil characteristics, vegetation cover, or human disturbances. Among CK, PA1, and PA2, CK still showed some separation from the other two points, indicating that, despite the overall similarity in community background, the CK sample point was still influenced by certain specific biotic or abiotic factors, leading to deviations in its community structure.

### 3.8. Inter-Species Differences of Soil Microbial Dominant Species

**Figure 8:**
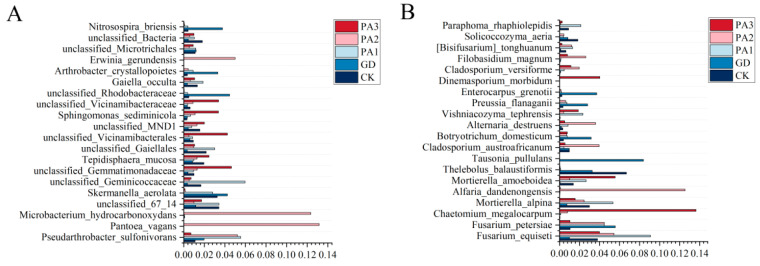
Inter-species differences of soil microbial dominant species. (**A**) The interspecific differences of the dominant bacterial species in the soil. (**B**) The interspecific differences of the dominant fungal species in the soil.

Inter-species analysis was conducted on the dominant microbial species in the soil across different sampling sites (Figure 8). The results indicated that the relative abundances of the 20 dominant bacterial species varied among the sampling sites (Figure 8A). The abundances of *Microbacterium hydrocarbonoxydans*, *Pantoea vagans*, and *Erwinia gerundensis* were significantly higher at the PA2 site compared to the other sites. The abundance of *Pseudarthrobacter sulfonivorans* was significantly higher at the PA1 and PA2 sites than at the other sites. In contrast, the abundance of *Skermanella aerolata* was significantly lower at the PA2 and PA3 sites compared to the other sites. The abundance of *Sphingomonas sediminicola* was significantly higher at the PA3 site, while the abundances of *Arthrobacter crystallopoietes* and *Nitrosospira briensis* were significantly higher at the GD site compared to the other sites.

In the fungal community (Figure 8B), the abundance of *Fusarium equiseti* was significantly higher at the PA1 site and significantly lower at the GD site compared to the other sites. The abundances of *Chaetomium megalocarpum* and *Dinemasporium morbidum* were significantly higher at the PA3 site. The abundances of *Alfaria dandenongensis*, *Cladosporium austroafricanum*, *Alternaria destruens*, and *Filobasidium magnum* were significantly higher at the PA2 site. Meanwhile, the abundances of *Tausonia pullulans*, *Botryotrichum domesticum*, *Preussia flanaganii*, and *Enterocarpus grenotii* were significantly higher at the GD site compared to the other sites.

In summary, the spatial distribution of dominant fungal and bacterial species in the soil exhibited strong habitat specificity. The unique soil physicochemical properties, nutrient availability, and microenvironmental differences at each sampling site actively shaped the microbial community structures through directional selection, with corresponding functional adaptations. This distribution pattern not only reveals the key filtering role of environmental factors in shaping microbial community assembly but also suggests that microorganisms in different ecological niches may undertake specialized ecological functions, such as organic matter degradation, pollutant transformation, nutrient cycling, and plant interactions.

### 3.9. Correlation Between Soil Physicochemical Properties and Bacterial Community Structure

**Figure 9:**
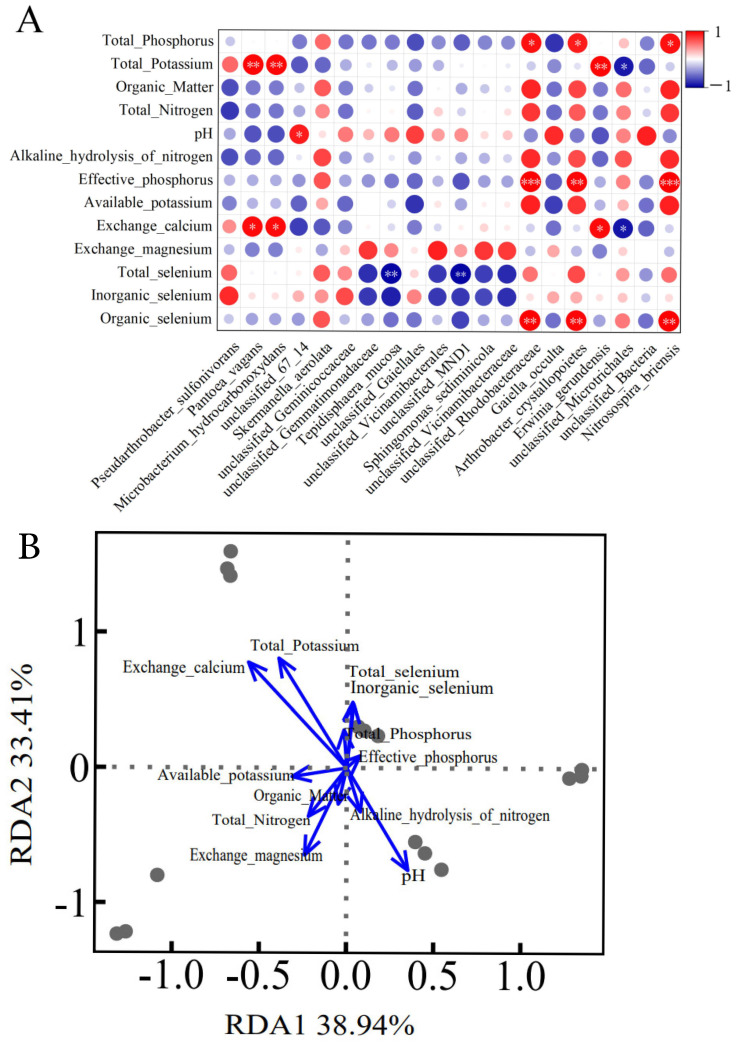
A correlation between soil physicochemical properties and bacterial community structure. (**A**) A correlation heatmap between soil physicochemical properties and the top ten dominant bacterial species (* *p* < 0.05, ** *p* < 0.01, *** *p* < 0.001). (**B**) A redundancy analysis (RDA) plot between physicochemical properties and bacterial community structure.

By analyzing the correlation between the physicochemical properties of different soils and their bacterial community structures (Figure 9), the results indicate that the abundance of specific bacterial groups is significantly associated with certain soil factors. As shown in Figure 9A, there are 16 positive correlations and 4 negative correlations among them. *Nitrosospira briensis* was significantly positively correlated with organic selenium (*p* < 0.01, r = 0.99), significantly positively correlated with effective phosphorus (*p* < 0.001, r = 1.00), and significantly positively correlated with total phosphorus (*p* < 0.05, r = 0.93). *Arthrobacter crystallopoietes* was significantly positively correlated with organic selenium (*p* < 0.01, r = 0.97), significantly positively correlated with effective phosphorus (*p* < 0.01, r = 0.96), and significantly positively correlated with total phosphorus (*p* < 0.05, r = 0.89). *Tepidisphaera mucosa* was significantly negatively correlated with total selenium (*p* < 0.01, r = −0.97). *Microbacterium hydrocarbonoxydans* was significantly positively correlated with exchangeable calcium (*p* < 0.05, r = 0.95) and significantly positively correlated with total potassium (*p* < 0.01, r = 0.95). *Pantoea vagans* was significantly positively correlated with exchangeable calcium (*p* < 0.05, r = 0.95) and significantly positively correlated with total potassium (*p* < 0.01, r = 0.97).

Figure 9B presents the visualization results of the relationship between soil physicochemical properties and bacterial community structure based on redundancy analysis. RDA analysis indicates that the soil physicochemical factors collectively explained 99.78% of the variation in the bacterial community. Among the measured soil factors, total potassium contributed the most (30.52%), followed by total nitrogen (21.47%), total phosphorus (15.07%), effective phosphorus (11.88%), and organic matter (11.50%). These five factors together accounted for 90.44% of the explanatory power, representing the primary drivers of the variation in bacterial community structure. RDA1 explained 38.94% of the community variation, while RDA2 explained 33.41%. RDA1 and RDA2 collectively explained 72.35% of the community variation, with total potassium, total nitrogen, total phosphorus, effective phosphorus, and organic matter being the key soil factors dominating the changes in bacterial community structure.

### 3.10. Correlation Between Soil Physicochemical Properties and Fungal Community Structure

**Figure 10:**
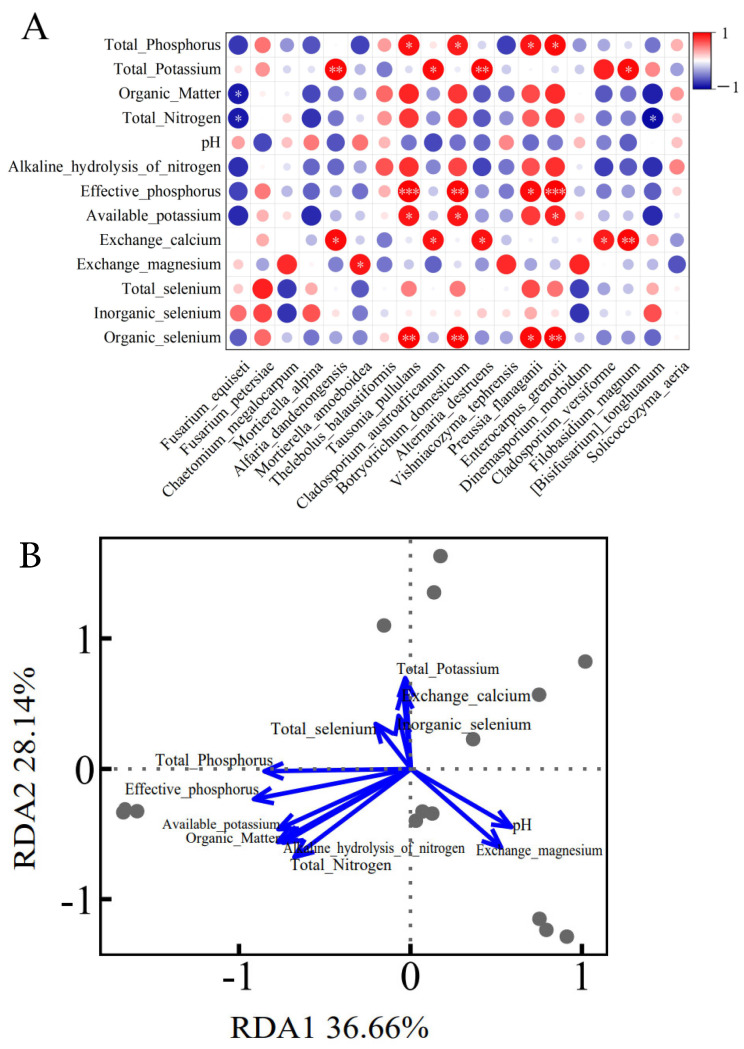
A correlation between soil physicochemical properties and fungal community structure. (**A**) A correlation heatmap between soil physicochemical properties and the top ten dominant fungal species (* *p* < 0.05, ** *p* < 0.01, *** *p* < 0.001). (**B**) A redundancy analysis (RDA) plot between physicochemical properties and fungal community structure.

Through the correlation analysis of the physicochemical properties of different soils and their fungal community structures (Figure 10), the results indicate that the abundance of specific fungal groups was significantly associated with certain soil factors. As shown in Figure 10A, there are 25 positive correlations and 3 negative correlations among them. *Enterocarpus grenotii* was significantly positively correlated with organic selenium (*p* < 0.01, r = 0.99), significantly positively correlated with available potassium (*p* < 0.05, r = 0.88), effective phosphorus (*p* < 0.001, r = 1.00), and total phosphorus (*p* < 0.05, r = 0.94). *Preussia flanaganii* was significantly positively correlated with organic selenium (*p* < 0.05, r = 0.96), effective phosphorus (*p* < 0.05, r = 0.95), and total phosphorus (*p* < 0.05, r = 0.92). *Botryotrichum domesticum* was significantly positively correlated with organic selenium (*p* < 0.01, r = 0.99), available potassium (*p* < 0.05, r = 0.91), effective phosphorus (*p* < 0.01, r = 0.98), and total phosphorus (*p* < 0.05, r = 0.90). *Tausonia pullulans* was significantly positively correlated with organic selenium (*p* < 0.01, r = 0.99), available potassium (*p* < 0.05, r = 0.90), effective phosphorus (*p* < 0.001, r = 1.00), and total phosphorus (*p* < 0.05, r = 0.94). In addition, all of *Filobasidium magnum*, *Alternaria destruens*, *Cladosporium austroafricanum*, and *Alfaria dandnongensis* were significantly positively correlated with exchangeable calcium (*p* < 0.05) and total potassium (*p* < 0.05).

Figure 10B presents the visualization results of the relationship between soil physicochemical properties and fungal community structure based on redundancy analysis. RDA analysis indicates that the soil physicochemical factors collectively explained 98.59% of the variation in the fungal community. Among the measured soil factors, total phosphorus contributed the most (27.30%), followed by total potassium (21.70%), total nitrogen (14.86%), organic matter (12.65%), and effective phosphorus (11.40%). These five factors together accounted for 87.11% of the explanatory power, representing the primary drivers of the variation in the fungal community structure. RDA1 explained 36.66% of the community variation, while RDA2 explained 28.14%. This further indicates that RDA1 and RDA2 collectively explained 64.80% of the community variation, with total phosphorus, total potassium, total nitrogen, organic matter, and effective phosphorus being the key soil factors dominating the changes in fungal community structure.

## 4. Discussion

An appropriate amount of selenium content in soil is beneficial for plant growth and plays a significant role in soil health and ecosystem stability [19,27]. The soil selenium content in the Aksu Prefecture of Xinjiang ranges from 0.11 to 0.89 mg/kg, with a mean value of 0.32 mg/kg, which can serve as a regional background reference [13]. The average soil Se concentration across Jiangxi was 0.44 mg/kg, exceeding both global (0.15 mg/kg) and Chinese (0.29 mg/kg) averages [14]. The mean total Se concentrations in cultivated soil samples were 1753.6 ± 742.8 µg/kg in Yutangba Village, Enshi City, China [40]. In our study, the soil selenium content in Ping’an district of Qinghai-Tibet Plateau ranges from 0.62 to 0.73 mg/kg, with a mean value reaching 0.68 mg/kg, which is significantly higher than this background level. Furthermore, selenium in the soil was predominantly present in the inorganic form, accounting for an average of 91% of the total selenium. This is different from the reported result that organic selenium predominates in the high-selenium soil in Yuxi, Yunnan Province [41]. This discrepancy primarily stems from significant differences in the parent rock materials and soil properties between the two regions, with the latter also being influenced by artificial measures such as biochar application.

Phosphorus application enhanced Se-rich lateritic red soil selenium supply capacity and the yield of selenium-rich agricultural products [42]. A significant amount of selenium in the soil of the Kohala region in Hawaii is closely related to organic matter [17]. The content of P in Ninghua County of Fujian Province is the main factor influencing the migration and transformation of Se in the soil–rice system [43]. In the selenium-rich soil of Shaanxi Province, the bioavailability of selenium can be regulated by adjusting the pH level and organic matter content of the soil [44]. The soil bulk density (BD), soil porosity, pH, cation exchange capacity (CEC), and electric conductivity (EC) values at five depth increments (0–20, 20–40, 40–60, 60–80, and 80–100 cm) in the Liupan Mountains in China showed a significant variation [45]. In our study, significant positive correlations between soil organic selenium content and total phosphorus (*p* < 0.05), effective phosphorus (*p* < 0.01) were shown.

Microorganisms can alter the chemical form and bioavailability of Se [46], and selenium can also attract microorganisms that promote plant growth, thereby enhancing the synergistic effect between plants and the rhizosphere microbial community [47]. Total phosphorus and available phosphorus are emerging as key factors influencing microbial diversity in the rhizosphere soil of Cymbidium tracyanum [48]. Dynamic changes in soil pH, total phosphorus content, and phosphatase activity significantly affect fungal diversity and the complexity of functional communities in the temperate rainforest region of New Zealand [49]. Nitrate-N, ammonium-N, and microbial biomass N concentrations had a large impact on soil bacterial communities, whereas nitrate-N and ammonium-N, available P, soil organic C, and microbial biomass C concentrations had a greater effect on soil fungal communities in the southwestern region of China [50]. Regarding the fungal community, the Shannon index and Chao 1 index of fungi in the rhizosphere soil of Sophora japonica at high altitudes both increase with the increase in altitude [51]. *Aspergillus niger*, an important fungus belonging to Ascomycota, can effectively influence the distribution and mobility of selenium [52]. In our study, the Ascomycota phylum was the dominant group. The Shannon index at the highest altitude point, PA3, was the highest, but the Shannon indices at the other sampling points were similar, which might be related to the fact that the study by Yu L was conducted in an area with a larger altitude gradient and more continuous changes in environmental factors. As for the bacterial community, Actinobacteria and Proteobacteria are the main dominant phyla in selenium-rich soils in Citrus rootstock soil [25] and the rich-selenium soil of Jiangtang Town, Jinhua City, Zhejiang Province [53]. *Rhodanobacter* and *Nitrospira* were predominant in the high-selenium ecosystem in Enshi City, Hubei Province, China [54]. In our study, Actinobacteria and Proteobacteria were the dominant bacterial groups in the soil, with Actinobacteria occupying the absolute dominant position. The selenium element in the rhizosphere soil of rape plants has increased the diversity of the microbial community and promoted the relative abundance of root-associated bacteria in the rhizosphere, such as Bryobacter, Nitrospirae, Rhizobiales, Xanthobacteraceae, Nitrosomonadaceae, and Basidiomycota [55]. In subtropical selenium-rich soil, SPB SynCom significantly increased the bioavailable Se content of the soil by up to 68.7% [56]. In our study, organic selenium content showed significant positive correlations with *unclassified Rhodobacteraceae* (*p* < 0.01), *Arthrobacter crystallopoietes* (*p* < 0.01), and *Nitrosospira briensis* (*p* < 0.01); whereas total selenium content showed a significant negative correlation with *Tepidisphaera mucosa* (*p* < 0.01).

Our study initially clarified the crucial regulatory role of soil phosphorus content in the process of organic selenium accumulation. Nevertheless, considering the complexity of environmental factors, this correlation still needs to be independently verified on a broader ecological scale or through controlled experiments. Future research should focus on the exploration of functional microorganisms, especially the uncultured Rhodobacteraceae, Crystallobacterium, and Nitrosococcus identified in this study, for their isolation and purification. Further investigation into the physiological and biochemical mechanisms of these potential functional strains in selenium transformation is also necessary.

## 5. Conclusions

This study focused on the soil selenium content, physicochemical parameters, and microbial communities of the selenium-rich area in Tibetan Plateau. The total selenium contents in both the Ping’an and Guide areas were high, ranging from 624.56 to 727.48 µg/kg. The organic selenium content was significantly positively correlated with total phosphorus, effective phosphorus, and available potassium contents. Within the bacterial community, organic selenium content showed significant positive correlations with the abundance of *Arthrobacter crystallopoietes* and *Nitrosospira briensis*. Total selenium content was significantly negatively correlated with the abundance of *Tepidisphaera mucosa*. In the fungal community, organic selenium content was significantly positively correlated with the abundance of *Tausonia pullulans*, *Botryotrichum domesticum*, *Preussia flanaganii*, and *Enterocarpus grenotii*. Total potassium, total nitrogen, and total phosphorus were the top three important influencing factors in the fungal and bacterial community. These microorganisms and phosphorus content mainly promote the accumulation of organic selenium in the soil.

## Data Availability

The original contributions presented in this study are included in this article. Further inquiries can be directed to the corresponding author.

## References

[1] Qu L., Xu J., Dai Z., Elyamine A.M., Huang W., Han D., Dang B., Xu Z., Jia W. (2023). Selenium in Soil-Plant System: Transport, Detoxification and Bioremediation. J. Hazard. Mater..

[2] Bai S., Zhang M., Tang S., Li M., Wu R., Wan S., Chen L., Wei X., Feng S. (2024). Effects and Impact of Selenium on Human Health, A Review. Molecules.

[3] Razaghi A., Poorebrahim M., Sarhan D., Björnstedt M. (2021). Selenium Stimulates the Antitumour Immunity: Insights to Future Research. Eur. J. Cancer.

[4] Kieliszek M. (2021). Selenium. Adv. Food. Nutr. Res..

[5] Pyrzynska K., Sentkowska A. (2024). Selenium Species in Diabetes Mellitus Type 2. Biol. Trace Elem. Res..

[6] Kieliszek M., Serrano Sandoval S.N. (2023). The Importance of Selenium in Food Enrichment Processes. A Comprehensive Review. J. Trace Elem. Med. Biol..

[7] Alshammari M.K., Fatima W., Alraya R.A., Khuzaim Alzahrani A., Kamal M., Alshammari R.S., Alshammari S.A., Alharbi L.M., Alsubaie N.S., Alosaimi R.B. (2022). Selenium and COVID-19: A Spotlight on the Clinical Trials, Inventive Compositions, and Patent Literature. J. Infect. Public Health.

[8] Meng W., Li X.-X., Wu P. (2021). New Insights into Selenium Enrichment in the Soil of Northwestern Guizhou, Southwest China. Bull. Environ. Contam. Toxicol..

[9] Hossain A., Skalicky M., Brestic M., Maitra S., Sarkar S., Ahmad Z., Vemuri H., Garai S., Mondal M., Bhatt R. (2021). Selenium Biofortification: Roles, Mechanisms, Responses and Prospects. Molecules.

[10] Cheng L., Nazir N., Li X., Zhang J., Zhu Y. (2025). Spatial Distribution of Surface Soil Selenium and Its Influencing Factors in China: A Literature-Based Analysis. Environ. Pollut..

[11] Zhang Y.-F., Ji B.-Y., Shen X., Yao Z., Ma Q. (2022). Formation mechanisms and significance of saline-lacustrine Se-rich soils in the Xining Basin. Geophys. Geochem. Explor..

[12] Zhang Y.-F., Dai L., Qin Y.-Q., He L.-Z. (2024). Opportunity analysis of the selenium-rich industry in assisting the construction of the “Four Places”. Qinghai Land Resour. Strategy.

[13] Zhai H., Zhang Y., Xu W., Hou W., Tang G., Ge C., Shao H., Gong H., Wang Y., Liu Y. (2024). Prediction and Pathway Models for Assessing Soil Properties Influencing Soil Selenium Enrichment and Bioavailability in Aksu Prefecture, Northwest China. Sci. Total Environ..

[14] Jiang F., Wu Y., Islam M.U., Jiang X., Wang B., He S., Lin X., Sun Y., Chen G., Chen X. (2024). Selenium Levels in Soil and Tea as Affected by Soil Properties in Jiangxi Province, China. BMC Plant Biol..

[15] Li J.-j., Yang T., Wang M.-g., Lian S. (2023). Characteristics and Influencing Factors of Soil and Crops Selenium Content in Eastern Sanjiang Plain. Environ. Sci..

[16] Li Y.-c., Liu J.-f., Li X.-z., Zhang D., Chen G.-d. (2023). Selenium Occurrence Characteristics and Bioavailability of Soil in the Hinterland of the Hetao Plain. Environ. Sci..

[17] Tolu J., Bouchet S., Helfenstein J., Hausheer O., Chékifi S., Frossard E., Tamburini F., Chadwick O.A., Winkel L.H.E. (2022). Understanding Soil Selenium Accumulation and Bioavailability through Size Resolved and Elemental Characterization of Soil Extracts. Nat. Commun..

[18] Tsioubri M., Gasparatos D., Economou-Eliopoulos M. (2020). Selenium Uptake by Lettuce (*Lactuca sativa* L.) and Berseem (*Trifolium alexandrinum* L.) as Affected by the Application of Sodium Selenate, Soil Acidity and Organic Matter Content. Plants.

[19] Wang Z., Huang W., Pang F. (2022). Selenium in Soil–Plant-Microbe: A Review. Bull. Environ. Contam. Toxicol..

[20] Fu R., Zhu M., Zhang Y., Li J., Feng H. (2025). Harnessing the Rhizosphere Microbiome for Selenium Biofortification in Plants: Mechanisms, Applications and Future Perspectives. Microorganisms.

[21] Wang Y., Hu C., Wang X., Shi G., Lei Z., Tang Y., Zhang H., Wuriyanghan H., Zhao X. (2023). Selenium-Induced Rhizosphere Microorganisms Endow Salt-Sensitive Soybeans with Salt Tolerance. Environ. Res..

[22] Han C., Cheng Q., Du X., Liang L., Fan G., Xie J., Wang X., Tang Y., Zhang H., Hu C. (2024). Selenium in Soil Enhances Resistance of Oilseed Rape to *Sclerotinia sclerotiorum* by Optimizing the Plant Microbiome. J. Exp. Bot..

[23] Li D., Zhou C., Wu Y., An Q., Zhang J., Fang Y., Li J.-Q., Pan C. (2022). Nanoselenium Integrates Soil-Pepper Plant Homeostasis by Recruiting Rhizosphere-Beneficial Microbiomes and Allocating Signaling Molecule Levels under Cd Stress. J. Hazard. Mater..

[24] Han X., Doménech-Pascual A., Donhauser J., Zohner C.M., Mo L., Crowther T.W., Casas-Ruiz J.P., Jordaan K., Ramond J.-B., Romaní A.M. (2025). Fungal Diversity as a Key Driver of Soil Multifunctionality along a European Latitudinal Gradient. Geoderma.

[25] Tang Y., Zhou Y., Wang P., Ge L., Lou W., Yan X., Li S., Wang X., Hu C., Zhao X. (2024). Selenium-Mediated Shaping of Citrus Rhizobiome for Promotion in Root Growth and Soil Phosphorus Activation. J. Agric. Food Chem..

[26] Wang Y., Shi X., Huang X., Huang C., Wang H., Yin H., Shao Y., Li P. (2022). Linking Microbial Community Composition to Farming Pattern in Selenium-Enriched Region: Potential Role of Microorganisms on Se Geochemistry. J. Environ. Sci..

[27] Jiang Z., Wang Z., Zhao Y., Peng M. (2024). Unveiling the Vital Role of Soil Microorganisms in Selenium Cycling: A Review. Front. Microbiol..

[28] Zhu D., Niu Y., Fan K., Zhang F., Wang Y., Wang G., Zheng S. (2021). Selenium-Oxidizing *Agrobacterium* sp. T3F4 Steadily Colonizes in Soil Promoting Selenium Uptake by Pak Choi (*Brassica campestris*). Sci. Total Environ..

[29] Guo J., Luo X., Zhang Q., Duan X., Yuan Y., Zheng S. (2024). Contributions of Selenium-Oxidizing Bacteria to Selenium Biofortification and Cadmium Bioremediation in a Native Seleniferous Cd-Polluted Sandy Loam Soil. Ecotoxicol. Environ. Saf..

[30] Li J., Liu R., Wu B., Zhang C., Wang J., Lyu L., Tong X., Wu F. (2022). Influence of *Arbuscular mycorrhizal* Fungi on Selenium Uptake by Winter Wheat Depends on the Level of Selenate Spiked in Soil. Chemosphere.

[31] Jiang D., Yu F., Huang X., Qin H., Zhu Z. (2023). Effects of Microorganisms on Soil Selenium and Its Uptake by Pak Choi in Selenium-Enriched Lateritic Red Soil. Ecotoxicol. Environ. Saf..

[32] Zhang Z., Kau M., Zang H., Wang Y., Duan Y., Zhang L., Liu Y., Yuan L. (2025). Selenium Regulated the Responses of Soil Bacterial Communities to Short-Term Elevated Atmospheric CO_2_ Stress. Environ. Res..

[33] Ma Y., Liu Q., Qiu Y., Ji B., Miao G., Huang B. (2018). Study on the Geochemical Characteristics and Genesis of Soil Selenium in Xining and Its Surrounding Areas of Qinghai Province. Comput. Tech. Geophys. Geochem. Explor..

[34] Soil SubCenter, National Earth System Science Data Center, National Science & Technology Infrastructure of China. http://soil.geodata.cn.

[35] (2014). Eco-Geochemical Evaluation of Analysis of Animal and Plant Samples—Part 2: Determination of Selenium Content—Atomic Fluorescence Spectrometry.

[36] Gao X.-P., Peng Y., Li F.-C. (2021). Monitoring the Migration of Selenium in *Camellia oleifera* Forest in Southern Jiangxi by Atomic Fluorescence Spectrometry. Chem. Eng. Des. Commun..

[37] Bao S.D. (2000). Soil and Agricultural Chemistry Analysis.

[38] (2006). Soil Testing—Part 13: Method for Determination of Soil Exchangeable Calcium and Magnesium.

[39] (2021). Delimitation and Identification of Naturally Selenium-Rich Land.

[40] Long Z., Xiang J., Song J., Lu Y., Yin H., Zhu Y., Liu X., Qin L., Bañuelos G.S., Wang Z. (2020). Soil Selenium Concentration and Residents Daily Dietary Intake in a Selenosis Area: A Preliminary Study in Yutangba Village, Enshi City, China. Bull. Environ. Contam. Toxicol..

[41] Tang Z., Feng X., Li R., Fan F., Miao Z. (2025). Mechanisms of Biochar in Modulating Soil Organic Selenium Transformation and Enhancing Soil Selenium Availability. Agronomy.

[42] Huang J., Huang X., Jiang D. (2024). Phosphorus Can Effectively Reduce Selenium Adsorption in Selenium-Rich Lateritic Red Soil. Sci. Total Environ..

[43] Wang Y., Yang Z., Chen G., Zhan L., Zhang M., Zhou M., Sheng W. (2023). Influencing Factors of Selenium Transformation in a Soil–Rice System and Prediction of Selenium Content in Rice Seeds: A Case Study in Ninghua County, Fujian Province. Environ. Sci. Pollut. Res..

[44] Liu N., Wang M., Zhou F., Zhai H., Qi M., Liu Y., Li Y., Zhang N., Ma Y., Huang J. (2021). Selenium Bioavailability in Soil-Wheat System and Its Dominant Influential Factors: A Field Study in Shaanxi Province, China. Sci. Total Environ..

[45] Rahman M., Zhang K., Wang Y., Ahmad B., Ahmad A., Zhang Z., Khan D., Muhammad D., Ali A. (2024). Variations in Soil Physico-Chemical Properties, Soil Stocks, and Soil Stoichiometry under Different Soil Layers, the Major Forest Region Liupan Mountains of Northwest China. Braz. J. Biol..

[46] Yang D., Hu C., Wang X., Shi G., Li Y., Fei Y., Song Y., Zhao X. (2021). Microbes: A Potential Tool for Selenium Biofortification. Metallomics.

[47] Ding M., Osayande I.S., Tsuda K. (2024). Selenium Nanoboosting of Plant-Beneficial Microbiome. Cell Host Microbe.

[48] Xie W., Tang Y., Li H., Dang M., Ci J., Zheng M., Zhang E., Wang Z. (2025). Physicochemical Properties and Microbial Community Structure of the Rhizosphere Soil of *Cymbidium tracyanum*. Front. Microbiol..

[49] Dunfield K.E., Mitter E.K., Richardson A.E., Gaiero J.R., Khosla K., Chen X., Wells A., Haygarth P.M., Condron L.M. (2024). Differential Structure and Function of Phosphorus-mineralizing Microbial Communities in Organic and Upper Mineral Soil Horizons across a Temperate Rainforest Chronosequence. Environ. Microbiol..

[50] Wang P., Xie W., Ding L., Zhuo Y., Gao Y., Li J., Zhao L. (2023). Effects of Maize–Crop Rotation on Soil Physicochemical Properties, Enzyme Activities, Microbial Biomass and Microbial Community Structure in Southwest China. Microorganisms.

[51] Yu L., Zhang Z., Liu P., Zhou L., Tan S., Tang C., Li Y. (2024). Elevated Altitude and Limestone Soil Promoted Fungal Diversity in Rhizosphere Soil of *Sophora japonica*. Horticulturae.

[52] Farkas B., Vojtková H., Bujdoš M., Kolenčík M., Šebesta M., Matulová M., Duborská E., Danko M., Kim H., Kučová K. (2021). Fungal Mobilization of Selenium in the Presence of Hausmannite and Ferric Oxyhydroxides. J. Fungi.

[53] Yue S., Feng C., Yang Y., Chen L., Guo Y. (2020). Analysis of microbial community structure and diversity in selenium-sand melon soil under different continuous cropping years. Agric. Res. Arid Areas.

[54] Sun Y., Guo J., Wei F., Chen X., Li M., Li C., Xia S., Zhang G., You W., Cong X. (2023). Microbial Functional Communities and the Antibiotic Resistome Profile in a High-Selenium Ecosystem. Chemosphere.

[55] Liu K., Cai M., Hu C., Sun X., Cheng Q., Jia W., Yang T., Nie M., Zhao X. (2019). Selenium (Se) Reduces Sclerotinia Stem Rot Disease Incidence of Oilseed Rape by Increasing Plant Se Concentration and Shifting Soil Microbial Community and Functional Profiles. Environ. Pollut..

[56] Feng Z., Sun H., Qin Y., Zhou Y., Zhu H., Yao Q. (2023). A Synthetic Community of Siderophore-Producing Bacteria Increases Soil Selenium Bioavailability and Plant Uptake through Regulation of the Soil Microbiome. Sci. Total Environ..

